# “Nothing to lose and the possibility of gaining”: a qualitative study on the feasibility and acceptability of registry-based randomised controlled trials among cancer patients and clinicians

**DOI:** 10.1186/s13063-023-07109-2

**Published:** 2023-02-07

**Authors:** Khic-Houy Prang, Bill Karanatsios, Angela Zhang, Ebony Verbunt, Hui-li Wong, Vanessa Wong, Lucy Gately, Ben Tran, Peter Gibbs, Margaret Kelaher

**Affiliations:** 1 Centre for Health Policy, Melbourne School of Population and Global Health, The University of Melbourne, Parkville, VIC Australia; 2 Department of Surgery, The University of Melbourne, Parkville, VIC Australia; 3 Western Health Chronic Disease Alliance, Western Health, St Albans, VIC Australia; 4 Personalised Oncology Division, The Walter and Eliza Hall Institute of Medical Research, Parkville, VIC Australia; 5 Department of Medical Biology, The University of Melbourne, Parkville, VIC Australia; 6 Department of Medical Oncology, Peter MacCallum Cancer Centre, Melbourne, VIC Australia; 7 Department of Medical Oncology, Ballarat Health Services, Ballarat, VIC Australia; 8 Department of Medical Oncology, Western Health, Sunshine, VIC Australia

**Keywords:** Registry-based randomised controlled trials, Oncology, Qualitative study

## Abstract

**Background:**

Randomised controlled trials (RCTs) are considered the “gold standard” for evaluating the effectiveness of interventions in clinical research. However, conventional RCTs are typically complex, expensive, and have narrow eligibility criteria, which limits generalisability. Registry-based randomised controlled trials (RRCTs) are an alternative approach that integrates the internal validity of an RCT with the external validity of a clinical registry by recruiting real-world patients and leveraging an existing registry platform for data collection. As RRCT is a novel research design, there has been limited research on the feasibility and acceptability of RRCTs from the patients’ and trial team’s perspectives. This study aims to explore patients’, clinicians’, and study coordinators’ perspectives towards participation in and conduct of oncology RRCTs in Australia.

**Methods:**

Thirty-seven semi-structured interviews were conducted with 15 cancer patients, 15 clinicians, and 7 study coordinators. Interviews were audio-recorded and transcribed verbatim. The data were analysed using thematic analysis.

**Results:**

Three overarching themes were identified: (1) enablers and barriers to recruitment and enrolment of patients in RRCTs, (2) experiences of patients participating in RRCTs, and (3) recommendations for the implementation of future RRCTs. For patients, altruism and “trust in the clinician” were key reasons to participate in a RRCT. For clinicians and clinical trial coordinators, the RRCT study design was perceived as “simple and straightforward” but “less exciting” than RCTs. Competition from commercially sponsored RCTs poses challenges for investigator-led RRCTs recruitment, particularly if eligible patient numbers are low. There were limited impacts on patients’ treatment experiences and clinicians’ clinical workflow given that the RRCTs explored different standards of care. Recommendations to improve the enrolment of patients in RRCTs included generating greater buy-in from clinicians by increasing awareness of RRCTs via education initiatives and broader promotion of the “selling point” of RRCTs and providing monetary compensation to hospitals for enrolling patients.

**Conclusions:**

Whilst patients, clinicians, and study coordinators were generally supportive of RRCTs, several barriers to effective RRCT implementation in oncology were identified. Developing strategies to increase acceptance of the methodology by clinicians will help enhance the uptake of RRCTs in Australia and internationally.

**Supplementary Information:**

The online version contains supplementary material available at 10.1186/s13063-023-07109-2.

## Introduction

Randomised controlled trials (RCTs) have long been viewed as the gold standard for evaluating the effectiveness of interventions in clinical research [1, 2]. While conventional RCTs have high internal validity, they can be resource-intensive, complex, and costly to run, and often have strict inclusion and exclusion criteria that constrain generalisability [1–3]. Alternative approaches to conventional RCT design have been sought to help overcome these challenges. One such approach is the registry-based randomised controlled trial (RRCT), which has received substantial interest due to the potential to bridge the gap between RCTs and real-world clinical practice. RRCTs incorporate major elements of conventional RCTs such as patient stratification and randomisation [3] and are best suited for testing hypotheses involving already-available clinical interventions for which there are uncertainties about the optimal combination, sequence, or duration of standard-of-care treatment, or where multiple standard-of-care options exist [2]. Treatment arms can include standard-of-care drugs, devices, procedures, or surgical interventions where there is clinical equipoise regarding which approach is superior [4–6].

A clinical registry is defined as “an organised system that uses observational study methods to collect uniform data to evaluate specified outcomes for a population defined by a particular disease, condition, or exposure, and that serves predetermined scientific, clinical, or policy purposes” [7]. Clinical registries serve multiple purposes, including the following: to describe the course of disease and care patterns, understand variations in treatment and outcomes, determine clinical or cost-effectiveness of services and products, monitor safety, and measure quality of care [7, 8]. Most recently, clinical registries serve as a platform to conduct RRCTs. In RRCTs, eligible patients who are enrolled in a clinical registry are also recruited to participate in an RCT where key characteristics such as baseline medical history, treatment received and trial-related endpoints can all be collected in the clinical registry. Eligible patients can also be identified by a targeted search of the existing clinical registry population and then recruited. As part of an RRCT, patients are randomly allocated to a treatment that is considered one of two or more standard-of-care strategies, where the optimal approach is yet to be determined [9]. A randomisation module may also be incorporated within the clinical registry.

The RRCT study design offers several notable benefits compared to conventional RCTs, including efficiencies in recruitment and data collection, minimal monitoring and substantially lower costs. Using clinical registries, RRCTs can identify and recruit patients more efficiently, avoid duplicate data collection, and facilitate the completeness of follow-ups. The inclusion of real-world patients increases the likelihood that RRCT results are generalisable to the wider population [3]. By recruiting patients from registries, a wider and more representative range of treatment centres can be involved in RRCTs, including those not resourced or equipped to conduct RCTs. This is significant as the patient population and even treatment outcomes at major hospital centres may vary significantly from smaller or regional centres that do not have an established trial program. Some reasons include the availability of expert subspecialist clinicians with access to a wide range of other multidisciplinary expertise and highly motivated patients who are willing to travel to a major hospital centre to participate in a clinical trial [3].

Unlike conventional RCTs which are typically conducted in a highly controlled environment, involved novel interventions with frequent follow-up and monitoring of safety and efficacy for regulatory requirements, and specialised assessment of endpoint measures, RRCTs require minimal monitoring and regulatory oversight. RRCTs typically investigate the optimal use of various standard-of-care treatments with existing regulatory approval. Simple and established endpoints are generally included in the clinical registry. As such, no additional monitoring is required beyond the standard-of-care follow-up intervals and no additional data beyond what is already collected in the registry [3, 6, 10].

There are substantial cost savings from RRCTs, which are generally initiated and managed by non-commercial investigators (e.g. research institute or university, hospital institution, scientific group or society, cooperative research group, or a clinical investigator), compared to commercially sponsored or other well-funded RCTs [11]. Commercially sponsored RCTs are initiated and funded by a commercial entity (i.e. pharmaceutical or device company), with the purpose to generate the data required to substantiate the efficacy and safety of an investigative product toward the goal of commercial release and marketing [12]. Cost savings from RRCTs result from reductions in administrative and training costs associated with establishing infrastructure for study databases, personnel costs for capturing and monitoring data, and not conducting protocol-specific procedures outside of standard care [3].

There have been a number of interventional cardiology RRCTs that serve as landmark studies that are exemplars of the benefits of RRCTs [13, 14]. The TASTE trial was a multicenter, prospective, randomised, controlled, open-label clinical trial, with patients recruited from the national comprehensive Swedish Coronary Angiography and Angioplasty Registry. A total of 7,244 patients diagnosed with an ST-elevation myocardial infarction and earmarked to undergo percutaneous coronary intervention (PCI) were randomised to undergo manual thrombus aspiration followed by PCI or to PCI only. The primary endpoint was all-cause mortality at 30 days, which was captured through the registry. The TASTE trial included 60% of all patients referred for PCI in Sweden and Iceland during the study period and was able to be completed in less than 3 years at a >90% cost savings compared to a conventional RCT [15, 16].

### Cancer registry-based randomised controlled trials

In oncology practice, there are often multiple standard-of-care treatment options available without there being one clear superior approach for a specific patient. This is in part due to the lack of head-to-head studies of standard-of-care treatments, some of which are developed in overlapping time periods [3]. RRCTs, therefore, have the potential to evaluate the effectiveness of standard-of-care treatment options in routine clinical care, by enrolling large numbers of patients using a clinical registry to help address research questions of public health interest.

In an effort to broaden the portfolio of oncology trials on offer, novel clinical trial methodologies such as RRCTs are being developed and tested. Three RRCTs led by the Walter and Eliza Hall Institute of Medical Research are currently underway across three tumour streams, with a focus on determining optimal sequencing and duration of treatment through assessing appropriate efficacy endpoints [11]. The first trial, ALT-TRACC, examines the feasibility of a multi-centre prospective RRCT using The Treatment of Recurrent and Advanced Colorectal Cancer (TRACC) registry, focusing on the sequencing of doublet chemotherapy [17]. The second trial, EX-TEM, examines the impact of extending the duration of post-radiation chemotherapy, using Brain Registry Australia: Innovation and traNslation (BRAIN) [18]. The third trial, REAL-Pro, compares the effects of two novel hormonal agents where differences in efficacy have not been clearly established, using the Electronic castration-resistant Prostate cancer Australian Database (ePAD) registry [19]. Details of each of the RRCTs are described in Table 1:

**Table 1:** Summary of RRCTs

	ALT-TRACC	EX-TEM	REAL-Pro
**Primary objective**	To examine the feasibility of a multi-centre prospective RRCT evaluating alternating oxaliplatin and irinotecan doublet schedules versus continuous clinician choice doublet chemotherapy during initial treatment of metastatic colorectal cancer	To determine the overall survival impact of an additional six cycles of post-radiation temozolomide following the current standard of concurrent chemoradiation and six cycles of temozolomide	To compare the incidence of cognitive decline in elderly patients with metastatic castration-resistant prostate cancer (mCRPC) treated with abiraterone acetate versus enzalutamide
**Study design**	Phase II open-label, multi-centre, registry-based randomised controlled trial	Phase III open-label, multi-centre, registry-based randomised controlled trial	Phase IIII Prospective, open-label, multi-centre, registry-based randomised clinical trial
**Population**	Patients diagnosed with treatment-naïve metastatic colorectal cancer (mCRC) deemed by their treating clinician to be fit for doublet chemotherapy	Newly diagnosed patients with histologically confirmed glioblastoma (WHO grade IV) and radiologically stable or responding disease (as per RANO criteria) following concurrent chemoradiation and six cycles of post-radiation temozolomide	Men aged 75 years or older with mCRPC appropriate for treatment with enzalutamide or abiraterone acetate
**Randomisation and treatment arms**	Patients randomised in a 1:1 ratio to one of two treatment arms: alternating cycles of oxaliplatin and then irinotecan doublet chemotherapy (experimental arm) versus continuous doublet chemotherapy (control arm).	Patients randomised in a 1:1 ratio after completing concurrent chemoradiation and six cycles of post-radiation temozolomide: observation (control arm) versus six further cycles (experimental arm) of post radiation temozolomide chemotherapy.	Participants stratified according to previous docetaxel treatment (yes/no) and randomised in a 1:1 ratio to enzalutamide versus abiraterone acetate
**Primary endpoint**	Progression free survival	Overall survival	Cognitive function
**Secondary endpoints**	Efficacy and toxicity by collecting data from the TRACC registry	Progression free survival, adverse events and the necessity for temozolomide dose modification to be determined by data entered into the BRAIN registry	Depression, falls and serious adverse events determined by data recorded in the ePAD registry

*ALT-TRACC* Alternating oxaliplatin and irinotecan doublets versus continuous doublet chemotherapy in previously untreated metastatic colorectal cancer, *EX-TEM* Phase III Trial of Extended Temozolomide in Newly Diagnosed Glioblastoma, *REAL-Pro* Registry-based Study of Enzalutamide vs Abiraterone assessing cognitive function in Elderly Metastatic Castration-Resistant Prostate Cancer

### Objectives of study

RRCTs are a relatively novel research design. To our knowledge, there is no published literature on the feasibility and acceptability of RRCTs in oncology and other disease settings, including exploring the barriers and enablers to their acceptance by patients, study coordinators, and clinicians. This study aims to explore the perspectives of cancer patients, clinicians, and clinical trial/study coordinators (hereafter study coordinators) following their participation in the ALT-TRACC, EX-TEM, and REAL-Pro trials. Specifically, this study aims to (1) explore the feasibility and acceptability of RRCTs and (2) identify the barriers and enablers to effective RRCT implementation. In doing so, this study will aim to identify strategies for greater uptake of RRCTs in Australia and internationally.

## Methods

### Study design

This study used a qualitative approach to capture the experience and attitudes of cancer patients, clinicians, and study coordinators involved in the three oncology RRCTs. Semi-structured interviews with participants were conducted using an interview guide. The interview guide was designed to elicit an understanding of the feasibility and acceptability of RRCTs including the barriers and enablers to effective RRCT implementation. Participants were invited to make additional comments to ensure that all topics they wished to discuss were covered. The qualitative study is reported in line with guidelines set out in the consolidated criteria for reporting qualitative research (COREQ) [20] (Additional file 1: Appendix 1).

### Recruitment of participants

Total population sampling was used to identify and select eligible participants. This is a type of purposive sampling technique that involves examining the entire population that has a set of characteristics. Hence, all patients were eligible for an interview if they were invited to participate in one of the three RRCTs, regardless of whether they chose to participate or not in the RRCT. The inclusion and exclusion criteria for patients were reflective of the individual RRCT criteria (Additional file 2: Appendix 2). Where patients may have been unable to complete an interview due to language barriers, proxy interviews were conducted with a family member, with the patient’s permission. Clinicians and study coordinators were eligible for interview if they were involved with one of the three RRCTs.

Patients enrolled in one of the three RRCTs were asked by their treating clinician during a consultation whether they would like to participate in this study exploring their experiences with RRCTs. If they were agreeable, their contact details were passed on to the research team. A member of the research team then contacted the patients via telephone and invited them to participate in the study. Each patient received a $50 gift card as compensation for their time participating in the study. A list of names and email addresses of the clinicians and study coordinators involved in the RRCTs were provided to the research team by either the lead principal investigator, project manager, or clinical trial coordinator of each RRCT. Initial contact and subsequent follow-up with clinicians and study coordinators were made via email by a member of the research team. A total of 61 participants were approached, with 20 patients consenting to be contacted from 83 patients enrolled in the RRCTs, along with 31 clinicians, and 10 study coordinators. Thirty-seven participants agreed to be interviewed (61% response rate). In all cases, if the individual indicated a willingness to participate, a plain language statement and consent form were sent by email or post, and a date and time for the interview was arranged.

### Data collection

The interviews were undertaken between August 2019 and July 2021 by one researcher (KP). KP (PhD, BAppSc (Hons) in psychology, BA) is a female health services researcher, with experience in conducting mixed methods research. All participants were interviewed via telephone with notes collected following the interviews. Interviews for patients, clinicians, and study coordinators averaged 33 min (ranged 14 to 49), 34 min (ranged 21 to 55), and 28 min (ranged 18 to 37) in length, respectively. All interviews were audio-recorded with the participant's consent. Participants were offered the opportunity to review their transcripts prior to data analysis. Five participants accepted this offer, and no corrections were made.

### Data analysis

Interview recordings were transcribed verbatim by a professional transcription service and imported into QSR NVivo 12 for coding . Thematic analysis using a combination of inductive and deductive coding was used for identifying, analysing, and reporting themes within the data [21, 22]. Two researchers (KP and BK) independently analysed five interview transcripts using a coding guide developed from the structure of the interview guide. The initial codes were then compared and refined through discussion between the researchers. The remaining interview transcripts were then coded by one researcher (KP), and emergent codes were added as needed. For theme development and revision, similar codes were clustered together and subsequently collapsed into emergent themes. The researchers discussed the emergent themes identified from the data until a consensus was reached.

### Ethical considerations

Ethical approval for this study was granted by the Melbourne Health Human Research Ethics Committee (HREC/48122/MH-2018). Hospital site governance was granted by five sites and three clinicians’ private rooms across Victoria, New South Wales, and Queensland, Australia. Written consent was obtained from all participants prior to data collection to record and use their interview data.

## Results

### Participants

A total of 37 interviews were conducted with 15 patients (including 3 proxy interviews), 15 clinicians, and 7 study coordinators across the three RRCTs (Table 2). Only 1 patient who declined participating in the RRCT was interviewed and therefore was excluded from the thematic analysis. Three patients initially accepted for their contact details to be passed on to the research team but 2 declined to participate in the interview when contacted and one was not contactable.

**Table 2:** Categories of interview participants by RRCTs

	ALT-TRACC	EX-TEM	REAL-Pro	Total
**Patients**	11^a^	2	2	15
**Clinicians**	8	4	3	15
**Study coordinators**	5	2	0	7
**Total**	24	8	5	37

^a^All patients interviewed were enrolled in the RRCTs except for one

Table 3 shows the demographic characteristics of the participants interviewed. Patients were predominantly males aged 65 years and older, married, and receiving the pension. The sample was representative of those who participated in the RRCTs for sex but not age groups. Older patients aged 65 years and older were slightly over-represented (71% vs. 66%), whilst younger patients aged 25-44 years were under-represented in this study (0% vs. 9%). Clinicians were mostly females, aged between 35 and 64 years old, time in practice ranged widely, and most worked part-time in a public hospital located in metropolitan areas. Study coordinators were all females, aged between 25 and 44 years old, and had worked less than 9 years in the role in a public hospital located in metropolitan areas.

**Table 3:** Demographic characteristics of participants

	Patients (***n***=14)*	Clinicians (***n***=15)	Study coordinators (***n***=7)
**Sex**			
*Male*	10	6	
*Female*	4	9	7
**Age groups**			
*25–34*			4
*35–44*		9	2
*45–54*	1	4	1
*55–64*	3	2	
*65+*	10		
**Marital status**			
*Single*	2		
*Married/de facto*	8		
*Separated/divorced/widowed*	4		
**Household income**			
*<$100,000*	2		
*Pension/superannuation*	12		
**Years in practice**			
*0–4 years*		1	6
*5–9 years*		4	1
*10–14 years*		5	
*15–19 years*		1	
*20+ years*		4	
**Hospital type**^**a**^			
*Public*		6	6
*Private*		2	1
*Both*		7	
**Hospital location of RRCTs**			
*Metro*		14	6
*Regional/rural*		1	1
**Employment status**			
*Full-time*		6	
*Part-time*		9	

*excluded patient not enrolled in the RRCTs

^a^ clinicians’ workplace, not limited to RRCTs site location

### Themes

Three overarching themes were identified: (1) barriers and enablers to enrolment of patients in RRCTs; (2) experiences of participants in RRCTs, including patients, study coordinators, and clinicians, and; (3) recommendations for the implementation of future RRCTs. The overarching themes and subthemes are presented in Table 4. The themes reflect the aims of the study and the topics set out in the interview guide.

**Table 4:** Overarching themes by sub-themes and participants group

Overarching themes	Sub-themes		
	**Cancer patients**	**Clinicians**	**Study coordinators**
Recruitment and enrolment of patients in RRCTs	Facilitators • Altruism • Personal gain • Trust in clinicians	Facilitators • Benefits of RRCT study design compared to RCT “simple and straightforward” • Equipoise	Facilitators • Broad patient eligibility criteria
	Barriers • Limited understanding of RRCT study concept	Barriers • Considering patients’ best interest • Low eligible patient volume • Competing commercially sponsored trials • Specific research question/study design • Perceptions of RRCT as “less exciting” than commercially sponsored RCT	Barriers • Low eligible patient volume • Competing commercially sponsored trials
Experiences of participating in RRCTs	• Minimal impact on patients (planning/organisation) • Pleased with treatment and care received	• Minimal impact on clinical processes (identifying and recruiting patients, implementation and monitoring)	• “Less work” than RCTs as minimal monitoring, fewer specimens and data collection, and limited coordination required between units and departments
Recommendations for the implementation of future RRCTs	Patients • Education • Patients sharing their trial experiences with other eligible patients	Clinicians • Education and increasing awareness of RRCTs • Identifying “tractability”/“selling point” of RRCT Hospitals • Involving regional/rural and private hospitals to increase the patient pool and give opportunity to participate in trials • Financial incentives for hospitals • Building capacity and support at the sites (e.g. data entry support for clinicians) • Regular engagement and updates from RRCT principal investigator to sites	Clinicians • Champion clinician at each hospital site Hospitals • Building capacity and support at the sites (e.g. study coordinators to flag eligible patients prior to consultation) • Coordination and support across units and departments (e.g. day oncology department and pharmacy) • Regular engagement and updates from RRCT principal investigator to sites

#### Recruitment and enrolment of cancer patients in RRCTs

##### Enablers

For patients, the prospect of “nothing to lose and the possibility of gaining” was a determining factor to their participation. Furthermore, participation provided an opportunity “to help somebody else” in the future. In some cases, patients’ personal experiences involving a family member or friend that had died due to cancer motivated them to participate in the RRCTs. Despite their altruistic reasons for participating in an RRCT, patients appeared to have a limited understanding of the concept of RRCTs, with several patients unclear whether they were enrolled in an RRCT. Patients generally placed their trust and faith in their clinicians to know and do what is best for them as they were not the “expert”:

I’ve got pretty good faith in the doctors and that and go with what they think. All I know is I’ve got it [cancer]. I’m trying to help. Well, I follow their directions, can’t you? Can’t do much else. [ … ] Put it this way, I build houses for a living, then. I wouldn’t like somebody come and tell me how to put a house together when they’ve had no experience at all. I’m not going to walk up to the doctor and tell her how’s this I want it to have or I want it done. You’ve got to go with the experienced ones. Patient 7

Interestingly, patients were likely to trust clinical trials led by universities or research groups more than commercial entities because they perceived commercial entities could potentially obfuscate the results to generate revenues for a new product. Some patients were more comfortable with participating in the RRCTs because the drugs had regulatory approval compared to trials with experimental drugs, or if they were already familiar with the drugs on offer (as for EX-TEM). Patients were generally supportive of data being collected in a clinical registry for research purposes and to generate new knowledge for future treatment if the data were kept secure and confidential.

I would trust the universities for those sorts of trials more than I would trust the trial by a big pharmaceutical company. I would want all sorts of guarantees and such from a pharmaceutical company to try out their new whizzbang drugs. I’d be less likely to go into it than I would universities or research facilities trial. Patient 6

No [concerns]. You’ve got to have information. How can you go anywhere without information? You know, it’s a Computerworld now and all that stuff, but you still got to have information from a person or their body to research what you’re doing, and if it’s working. Otherwise, what’s the good? Patient 2

Clinicians and study coordinators were supportive of RRCTs given how “simple and straightforward” their study design was, and the anticipated minimal costs associated with conducting RRCTs as it leverages existing registry infrastructure to collect clinical data. Clinicians and study coordinators perceived RRCTs to have strong external validity due to the broad patient eligibility criteria compared to commercially sponsored RCTs and their ability to “address real-world questions using real-world data”. In particular, RRCTs were perceived by clinicians to be a “powerful tool” to address research questions where there was equipoise between standard-of-care treatments:

I think if you feel strongly that the patient for one reason or another should have one treatment regimen or another, then they shouldn’t be put on a trial that randomises them. The idea about a trial is equipoise you know. You as the researcher feel there’s a clinical question that needs to be answered and you believe that both arms of the study are equally weighted to provide benefits to the patient. If you truly believe that the patient should have one chemotherapy regimen over another, and that it would be detrimental to the patient to have a treatment that is offered in the study, then you shouldn’t put them on the study. Clinician 1

However, clinicians acknowledged that the internal validity of an RRCT was unlikely to be as robust as an RCT given the less intense oversight and monitoring. They also noted that the success of RRCTs was dependent on the registry capacity to collect long-term patient outcomes and toxicities accurately and in a timely manner. As such, having a robust data infrastructure to collect and clean the data with quality checks and adequate ongoing financial support were considered important factors for the long-term viability of the clinical registry:

I still think that there’s the element of uncertainty about the quality of the data. Do you know what I mean? That’s [registry data] been around for a long, long time or tracking in various incarnations. It’s been done very well, but any registry itself, the quality of the data needs to be verifiable. Clinician 13

##### Barriers

Given the benefits of RRCTs, clinicians expected that patient enrolment would be much faster than what was the case. However, several enrolment barriers were identified: variability in clinicians’ ability to recruit; patients’ suitability and capability to participate; low eligible patient volume at the site; competing commercially sponsored RCTs; and perception that RRCTs explore research questions that are “less exciting” than those of commercially sponsored RCTs.

There was variability in the approach to the recruitment process of eligible patients to participate in the RRCTs versus an RCT across clinicians. Some clinicians perceived it as their duty of care to disclose all open clinical trials including RRCTs then recommend which one was best suited to them after discussing the advantages and disadvantages of each trial with the patient:

No, that is extremely bad care to do that and it’s disrespecting the patient’s rights to actually know what all the options are before making a decision [ … ] You actually should. Legally you should. If you don’t, you could get yourself in a lot of trouble. If I had a series of trials for the same patient, we would discuss all of those trials and standard of care with that patient. Clinician 14

Other clinicians were unlikely to inform the patients of all open clinical trials as this was not feasible during a consultation and was perceived to be unfair to the patients and may impact their ability to make an informed choice as the description of multiple trial options may cause confusion and leave them overwhelmed. When selecting a clinical trial or several trials to discuss with patients, some clinicians considered the study research question/s including drug toxicities and the potential for improvement in health outcomes. Ultimately clinician trial preference was prefaced around what was in the patient’s “best interest” and their suitability and capacity to participate in the clinical trial:

I think that’s quite difficult. It’s very confusing for the patient, I think, to have a discussion about multiple possibilities. I think your role as a clinician is to try and work out for the patient in front of you which you think would be the best treatment option for them. So, I think you talk about the one that you think will have the best outcomes in terms of treating the cancer, but the toxicities, and also the impact on their lifestyle. So, some trials are very labour intensive and require multiple visits, not suitable for patients that live far away or have difficulty with transport. So, I think as a clinician you make the decision about, just as you make the decision about what chemotherapy treatment you think is right for them, you make the decision for that patient in front of you as well. Clinician 1

Low patient volume, in particular for a less common cancer such as brain cancer, was also considered to limit the number of eligible patients that clinicians encountered in their practices, restricting enrollment in an RRCT, despite the broad eligibility criteria. Competing commercially sponsored RCTs for the same pool of the cancer population also could impede clinicians’ ability to enrol patients in an RRCT. In some cases, clinicians would prioritise enrolling patients in a commercially sponsored RCT over investigator-led RRCT if it involved a “very exciting new drug” that was currently not available. Also, a consideration, was any impact on a patient’s eligibility for future clinical trials if they were to be enrolled in an RRCT. This is further compounded by the fact that RCTs may be a source of additional funding support to the hospitals, with some hospitals having recruitment quotas for commercially sponsored RCTs. Some clinicians noted that commercially sponsored RCTs provided an opportunity to subsidise investigator-led trials:

I guess the lack of funding is negative for the trial unit overall. If you’re going to do, we wouldn’t be able to run entirely registry trials, through our trial unit. The fact that these trials are not particularly funded, therefore, the pharma trials that we run subsidized these trials. I think they’re [RRCTs] important to run and they’re important questions to answer, but we couldn’t have a trial unit that just purely did studies such as these. There wouldn’t be any money to pay the staff. Clinician 12

There were some concerns among clinicians and study coordinators that the RRCT study research questions may be “less exciting” than commercially sponsored RCTs and therefore would not generate sufficient interest among clinicians for them to recruit patients, as RRCTs explored variations of standard care rather than novel drugs. Nonetheless, most clinicians conceded that the RRCT research study design was “going to be the way of the future” and provided an opportunity to address important clinical research questions that could inform clinical practice and were generally of no interest to commercial entities.

I think it’s a combination of some competing trials, I’ve already mentioned [competing trial name]. I think some people think the clinical questions are not that exciting. A lot of trials in oncology are only niche trials. They actually got this receptor, this mutation, whatever, but then they’re hard to do because you’re going to screen many, many people before you get one [eligible patient]. Clinician 9

#### Experiences of participating in RRCTs

##### Patients

Patients recalled receiving adequate information about the RRCTs and discussing the treatment options including potential side effects with their clinicians. A small proportion of patients sought additional advice and support from their spouse or other family members before participating in the RRCTs. Patients believed that they would have received the same or similar treatment regardless of whether they participated in the RRCT or not. As such, the variation in treatment on offer across the RRCTs made “no difference” to them, although some were unclear about how the randomisation to different arms occurred:

I’ve never really looked at it as being, okay I’m on the experimental trial sort of thing because I’m going for my treatment anyway which is not going to change. So, I just take it all on board as being okay, this is part of my regular fortnight [treatment]. Patient 1

Patients perceived no additional demands from the RRCTs beyond what they would have been required to do as part of their standard-of-care treatment including the commute to the hospitals and the time spent receiving treatment. Overall, patients had limited concerns about participating in RRCTs, besides drugs’ side effects which were expected but acknowledged this was not necessarily related to participating in the RRCT itself. They were generally pleased with the treatment and care received from their clinicians and hospital staff:

Okay. Because I was on it [chemotherapy] fortnightly, everything was explained thoroughly to make sure that I understood what drugs were going to be given, and how often, and all that sort of thing. So, I was really happy with being able to understand it. I think that was my main concern when I first started treatment, was I need to know what it was and any problems, just give them a call. Patient 8

##### Clinicians and study coordinators

Clinicians and study coordinators were satisfied with the amount of information provided about the RRCTs at the site initiation from the lead investigator of each of the RRCTs. This generally involved a site-initiation presentation and related documents about the study research aims, patients inclusion and exclusion criteria, enrolment and randomisation processes, treatment regimens, and data collection involved:

Well, there was a description of the study objectives and the inclusion or exclusion criteria. The description included the processes that are required to enroll patient and both a discussion regarding the planned recruitment and timelines for recruitment and how long the study would last, as well as the study-related procedures that patients would undergo and finally, practical information about recruitment of participants in terms of how to go about recruiting patients. Clinician 7

The process of identifying and recruiting eligible patients varied according to resources available at a hospital site and their current practices. In some hospitals, eligible patients were identified and discussed in weekly or fortnightly multidisciplinary team meetings involving oncologists, surgeons, and radiologists. In other hospitals, study coordinators provided additional support to the clinicians by identifying potentially eligible patients from a patient list prior to the consultations, with the caveat that the clinicians would verify the eligibility of the patients. In private rooms with no additional support, the onus was on the clinicians to identify and screen eligible patients during the consultations. Clinicians reported being able to easily identify eligible patients for the RRCTs based on the inclusion and exclusion criteria screening tool provided. This was noted to be simplest for EX-TEM given that the clinicians would have already established a rapport with their patients for 6 months prior to inviting them to participate in the RRCT. Clinicians felt confident providing information about the treatments to the patients, although they acknowledged that patients were unlikely to understand the concept of an RRCT:

Oh, yes. I think a lot of patients didn’t kind of really understand how it was a trial. So it was just talking to them about sequencing the chemotherapy regimens, and how that was an unanswered question. Most patients think of trials as receiving experimental drugs, so it was a different conversation. Clinician 1

Clinicians and study coordinators reported that the randomisation of patients into study arms, either in the clinic or off-site across the RRCTs, was relatively “straightforward”. Study coordinators were responsible for the randomisation of patients at the time of consent and notified the clinicians of the outcome to proceed with the drug prescriptions. There were no concerns with patients’ attrition given the study design, with disease progression more likely to contribute to attrition, but this would occur regardless of whether patients participated in the RRCT or not. Data collection and entry into the registry also varied across sites with clinicians entering the data themselves or with the assistance of a study coordinator or a data coordinator if available. Overall, clinicians perceived RRCTs to be “less work” than commercially sponsored RCTs but “more work” than standard of care:

Well, I think that as I said, from a clinician’s point of view, they’re [RRCTs] very straightforward. It is usually a practical question. In some ways there’s less monitoring, there’s less oversight, there’s less SAEs [seriously adverse events] and all the other rigmarole that goes with doing clinical trials these days. I certainly haven’t been bombarded with emails every several times a week, like I am for a lot of the other studies that I’m PI [principal investigator] on to ask me to go online and do this training and do that. I really appreciated the fact that as I said, it’s a very simple and straightforward design. Not too much work but still addressing the important clinical questions. I think I like the straightforwardness of it, the practicalities, and lack of ridiculous oversight which is becoming part and parcel of standard industry of sponsor trial. Clinician 9

Similarly, study coordinators were pleased with the simplicity and the minimal workload associated with RRCTs compared to commercially sponsored RCTs given it involved regulatory-approved drugs and required minimal coordination across various hospital units:

Not really [extra work], actually. It’s not something that I dread doing. It’s just something, just add on, like, I said it’s straightforward, simple data collection. Not as full on as the other workload that I have. Less people that I have to be in contact with, so for this one [RRCT] since it’s already an approved medication, I don’t have to do allocation medication and don’t really have to involve pharmacy as well the nurses. Not something extra workload, or what not, so it’s quite good. Study coordinator 2

#### Recommendations for the implementation of future RRCTs

Clinicians and study coordinators suggested the following strategies to increase patient recruitment into RRCTs and to improve the implementation of future RRCTs: (1) buy-in from clinicians by increasing awareness of RRCTs via education and identifying the “selling point” of RRCTs; (2) develop clinical research questions of interest to clinicians for RRCTs; (3) identify a clinician ‘champion’ at each site and provide resources to support the implementation of RRCTs including a study coordinator; and (4) offer financial incentives to sites for participating in RRCTs including opening RRCTs in regional/rural sites across Australia.

RRCTs were perceived to be a relatively novel concept in oncology practice and Australia. Clinicians and clinical trial coordinators proposed increasing awareness of RRCT study design by identifying its “selling point” and describing its advantages over RCTs for both clinicians and patients given that an RRCT does not involve an exciting new experimental drug. This could involve the dissemination of information via educational workshops/seminars to clinicians and clinical trial units. Dissemination of findings from RRCTs to the broader scientific community was considered important to showcase the applicability of RRCTs in oncology to address relevant clinical research questions:

I think it’s the [RRCT] concept that needs to be discussed a lot further. All clinicians appreciate the advantages. The more it’s spoken about, the more people are educated about it there’s probably about greater uptakes or being involved. I think I’ve got the advantage of hearing about it through my connections, but I’m not sure that all clinicians out there have a great understanding of them. Trying to educate people about them through presentations and meetings and things like that has been an advantage. It’s getting the message out a bit better. [ … ] I suppose when results of some of these studies come through, that’ll be a key opportunity to show the advantages and talk about the methodology and that sort of thing and try and roll out more of them. Clinician 10

The RRCT research question was perceived to be a key factor to increase engagement with clinicians to ensure ongoing participation in RRCTs. Given that RRCTs typically focus on variation of standard of care, the clinical questions that an RRCT may be able to address will be dependent on a number of factors including but not limited to the tumour type and staging, drug availability, and line of treatment previously or currently offered as standard of care. As such, some clinicians reported that RRCTs may be better suited for surgical and radiation trials where different techniques, as opposed to treatment protocols, are compared:

I think the most important thing is making sure we can randomise patients simply and we can show the data backbone. It all comes down with all research, did we ask the right question? If you don’t ask the question that’s going to be supported by everyone then you’re not going to get enrollment. The registry trials that we think it’s acting as trying to do things simple where you don’t need to involve trial pharmacies. You’re going to compare standards of care, the standard way of doing things. It’s maybe more suited to surgical and radiation trials and techniques, as opposed to new drugs because new drugs need trial pharmacy. If you’re going to involve all that, you might as well do a normal trial as opposed to registry trial. If we can compare two standards of care, if there’s a real question comparing standards of care, then it’s perfect for medical oncology trials. Unless you get that question right, it’s going to be hard to recruit [patients]. Clinician 7

Clinicians suggested that having a dedicated study coordinator to flag potential eligible patients from a clinic list and provide the required study documents (e.g. patient information consent form) to the clinicians prior to the consultation with patients would help increase patient enrolment in RRCTs. To minimise clinicians’ workloads, other supportive tasks and responsibilities perceived to be acceptable for study coordinators included: patient randomisation into arms, coordination with relevant units such as day oncology to inform patient allocation, and data entry into the clinical registry. For study coordinators, they reported that having an engaged principal investigator with regular updates about the RRCTs was important to remind the sites that their participation in an RRCT can potentially inform changes in the standard of care for patients in the future. Having a “champion” clinician on-site would also improve the visibility of the RRCTs and increase enthusiasm and motivation among staff to increase patient recruitment and enrolment:

I think mainly internally it would probably be more beneficial for us. As myself, as a study coordinator, it’d be more beneficial if I had a lot more support. I don’t feel like I do have the majority of support with the investigators and what not. They lack the drive, I suppose, because it’s not as exciting. I think if it comes from the investigator and works all the way down, then, yes, I think it would, like if they are more excited about it and pushing forward, then I think it would continue down through the whole team. Study coordinator 3

Given that commercially sponsored RCTs focused on large hospitals in metropolitan areas for patient recruitment, clinicians proposed involving regional and rural hospitals, private practice, and private hospitals in undertaking RRCTs to expand the pool of eligible patients and address the challenge of losing patient recruitment to competing commercially sponsored trials. This will enable clinicians and various sites to contribute to scientific research and build clinical trial capacity in-house. This will also provide an opportunity for patients outside of metropolitan areas to participate in RRCTs, thus reflecting a wider representation of real-world patients:

I think those populations of patients, because the people are different, their race is different, their age is different, some of these studies are European studies, some of the are Asian studies. Having a registry based Australian based study, with our population here, and including many patients that cannot be included in a standard randomised controlled trial, is a very valid, strong point. Clinician 2

Furthermore, unlike commercially sponsored RCTs where hospitals received large monetary compensation for enrolling patients, hospitals participating in RRCTs received minimal or no monetary compensation. This was perceived to be a concern for a trial unit as funding is required to support RRCT activities including staff remuneration. Clinicians acknowledged that although the RRCT activities were less onerous than an RCT, providing financial incentives was appropriate because it requires considerable effort to recruit and enrol patients and time to complete data entry in the clinical registry. As such, clinicians proposed that the monetary compensation for RRCTs should reflect the amount of work involved:

Well, we’re realistic about the fact that if you’re [RRCT investigators] not asking this at the same level of resource implications [commercially sponsored RCTs], so, yes, it’s a simple question and we don’t have to do as much work then we shouldn’t expect to be paid as much [ … ] yes, it’s important to put prices on trials, and the question is right, and we get some recompense for the work that we do, then it’s a win-win. Clinician 4

## Discussion

This qualitative study explored cancer patients’, clinicians’, and study coordinators’ perspectives of RRCTs in Australia. We found that RRCTs were acceptable to patients and they were generally supportive of participating for the greater good and on the recommendation of their treating clinician. However, several barriers to effective RRCT implementation in oncology were identified at the clinician level including lack of access to eligible patients, RRCTs not being ‘exciting’, and that commercially sponsored RCTs are a valued revenue source for hospitals.

Our study highlighted that personal and altruistic reasons, and trust in clinicians were important factors for patients’ participation in RRCTs. Our findings are consistent with a cross-sectional study which reported that patients with renal disease would be willing to participate in a hypothetical RRCT if it was “good for research” and “help someone else” [23]. These motivators are not unique to RRCTs and are common in RCTs, with patients more likely to participate in a trial if they trust their clinicians and perceived their clinicians as wanting them to join, highlighting the influence that clinicians can have on a patient’s decision-making [24–26]. Interestingly, we identified that patients favour and trust RRCTs because they are not commercially initiated and motivated. Given the trust that patients put in their clinicians’ advice, better educating clinicians on the benefits of RRCTs would be a worthwhile intervention to help increase the uptake of RRCTs by clinicians and improve patient recruitment.

There was evidence of poor understanding of the RRCT study design despite patients reporting that they received adequate study information. Several participants were unclear if they were enrolled in an RRCT and did not appear to understand the randomisation process. A lack of understanding among patients in relation to the details of the trial they are enrolled in is also a common finding among RCTs [25]. As the education level of patients can vary considerably, a greater emphasis on the acceptance of the potential risks of being involved in a study rather than an appreciation of the study design should be acceptable for informed consent when RRCTS involve standard-of-care interventions. As cancer afflicts predominantly the elderly, the level of understanding about a trial for the patient also needs to be tailored to their specific concerns and the information needs of their support team (family and friends). The RRCTs involved patients receiving variations of standard of care instead of novel drugs. This, therefore, did not impose any additional requirements or burden on the patient as compared to commercially sponsored RCTs. The lack of substantive deviation from standard care may also explain why certain patients were unclear if they were participating in an RRCT.

Clinicians and study coordinators identified several benefits of the RRCT study design compared to the RCT including leveraging existing registry infrastructure to collect clinical data, thereby minimising the financial costs of data collection. Furthermore, clinical equipoise was perceived as important by clinicians for a patient’s recruitment into an RRCT. This is consistent with previous research which identified a lack of equipoise between treatment arms as a barrier to RCT enrolment [27, 28]. Awareness of clinicians’ perceptions or preferences for particular treatments would be beneficial in ensuring any RRCT hesitancy or concerns are promptly addressed.

Despite the benefits of RRCTs, several barriers to enrolment were identified. Clinicians and study coordinators discussed how it was not feasible to inform patients of all open clinical trials due to time constraints, and that there was often a lack of eligible patients, particularly for rarer cancers. These barriers are also common in RCTs [27–29]. Using existing clinical registry data to identify the incidence of cancer type at each site prior to the conduct of RRCTs and involving multiple sites, especially for rare cancers, would be helpful to ensure sufficient numbers were recruited*.* Registry custodians could identify eligible patient*s* by notifying the treating clinicians or patients directly of available RRCTs if patient eligibility criteria are met [30]*.* Study coordinators could support clinicians by flagging eligible patients prior to consultation and by providing further information about the RRCT following confirmation of the patient’s eligibility from the clinicians.

Additionally, clinicians and study coordinators perceived RRCTs to be “less exciting” and not well funded compared to commercially sponsored RCTs, highlighting the need to promote the benefits of RRCT study design, and developing research questions alongside clinicians that will provide clarity to the efficacy of certain clinical practices. This will require active promotion, engagement, continued commitment, and advocacy from RRCT champions. Wouters et al. [31] proposed using a gradual learning healthcare system (LHS) approach to increase clinicians’ engagement in scientific research and to bridge the gap between research and standard of care. Shifting the culture among clinicians and incorporating real-time clinical feedback using comprehensive routine data collection are key in developing a LHS, with the goal of embedding comparative effectiveness trials such as RRCTs into standard-of-care delivery to create the ultimate full LHS.

Funding was identified as a major determinant of success for RRCTs, particularly for establishing and maintaining registries and the associated RRCT infrastructure required to conduct them and where they needed to exist alongside and at times compete against fully funded and well-supported commercially sponsored RCTs. RRCTs are unlikely to remunerate sites at the level of commercially sponsored RCTs, however, RRCTs are less resource-intensive and more aligned with routine clinical practice. It is anticipated that with greater awareness of RRCTs particularly among clinicians and study coordinators, the positive attributes of RRCTs should be compelling enough to allow them to be a viable alternative to commercially sponsored RCTs in certain circumstances.

### Limitations

To our knowledge, this is the first qualitative study to explore the feasibility and acceptability of RRCTs among patients, clinicians, and study coordinators in any disease type. As such, we were unable to compare our findings to previous research. Given the growth in RRCTs in oncology [32] and other diseases [6, 10], there is an opportunity to explore the design and implementation processes of RRCTs from the perspectives of patients, clinicians, and relevant stakeholders. Several limitations should be considered including selection bias. We interviewed only one patient who declined to take part in the RRCT, as the information was recorded by one clinician. We were unable to identify further similar patients as the registry did not capture those who were eligible but declined to participate in the RRCT. Interviewing these patients would provide a further understanding of why patients decline to participate in RRCTs, thereby enabling the development of appropriate strategies to improve patient recruitment. We proposed that future RRCTs collect such information in the registry. We interviewed only one clinician from a regional/rural hospital, so it is unclear whether the enablers and barriers identified in metropolitan hospitals are generalisable to regional/rural hospitals. Further research is warranted to evaluate the impact of the RRCTs in these settings. We also did not interview nurses and pharmacists who were responsible for the delivery of intravenous chemotherapy on-site as part of ALT-TRACC. Finally, this study was conducted during the COVID-19 pandemic, which may have impacted the enrolment of new patients into RRCTs, and subsequently patient recruitment for interviews.

## Conclusions

Whereby RCTs and RRCTs share common challenges concerning recruitment of patients, given that RRCTs are still a relatively unknown methodology in oncology and predominantly used for comparative effectiveness studies, they are currently not able to successfully compete against commercially sponsored RCTs for the same patient population. Commercially sponsored RCTs offer access to novel therapies with funding to adequately support the study which easily counteracts the identified enablers of RRCTs such as their simple study design and minimal impact on clinician workflow and patient time commitment. Creating greater awareness with patients and clinicians alike about the collective benefits of RRCTs via targeted education and promotion was identified as a key priority in our study findings to help maximise patient recruitment and address some misconceptions.

## Supplementary Information

**Additional file 1:** **Appendix 1.** COREQ checklist

**Additional file 2:** **Appendix 2.** Inclusion and exclusion criteria for cancer patients across the three trials.

## Data Availability

The datasets generated and analysed during the current study are not publicly available due to participants’ confidentiality.

## Abbreviations

ALT-TRACC: Alternating oxaliplatin and irinotecan doublets versus continuous doublet chemotherapy in previously untreated metastatic colorectal cancer
BRAIN: Brain Registry Australia: Innovation and traNslation
COREQ: COnsolidated criteria for REporting Qualitative research
EPAD: Electronic castration-resistant prostate cancer Australian Database registry
EX-TEM: Phase III Trial of Extended Temozolomide in Newly Diagnosed Glioblastoma
LHS: Learning healthcare system
PCI: Percutaneous coronary intervention
RCT: Randomised controlled trial
REAL-Pro: Registry-based Study of Enzalutamide vs Abiraterone assessing cognitive function in Elderly Metastatic Castration-Resistant Prostate Cancer
RRCT: Registry-based randomised controlled trial
TRACC: The Treatment of Recurrent and Advanced Colorectal Cancer registry
