# Supplementary material for: “Nothing to lose and the possibility of gaining”: a qualitative study on the feasibility and acceptability of registry-based randomised controlled trials among cancer patients and clinicians
Source: Trials. 2023 Feb 7;24:92. doi: 10.1186/s13063-023-07109-2 (PMC9902247; doi:10.1186/s13063-023-07109-2)
Supplement: Supplementary file 2 — Additional file 2: Appendix 2. Inclusion and exclusion criteria for cancer patients across the three trials. [file 13063_2023_7109_MOESM2_ESM.docx]

**Appendix 2 Inclusion and exclusion criteria for cancer patients across the three trials**

| **ALT-TRACC** | | **EX-TEM** | | **REAL-PRO** | |
| --- | --- | --- | --- | --- | --- |
| **Inclusion criteria** | **Exclusion criteria** | **Inclusion criteria** | **Exclusion criteria** | **Inclusion criteria** | **Exclusion criteria** |
| • Age ≥18 years  • Histologically confirmed, metastatic colorectal adenocarcinoma treated with ≤ 2 cycles of doublet chemotherapy  •  • ECOG performance status of 0-2  • Life expectancy of ≥3 months  • Adequate major organ function to receive doublet chemotherapy as judged by the treating clinician  • No contraindications to any of the 3 cytotoxic agents (5FU, oxaliplatin and irinotecan)  • Recent imaging of chest, abdomen and pelvis. It is recommended that this should be within 28 days of first chemotherapy dose (no more than 8 weeks) | • Previous chemotherapy and/or biologic therapy for CRC, except for adjuvant treatment if completed more than 6 months earlier  • Not suitable for doublet chemotherapy, as judged by the treating clinician  • Significant concomitant medical condition which the treating clinician believes precludes the patient from enrolling in the study | • Newly diagnosed, histologically confirmed glioblastoma  • Adults, aged ≥ 18 years  • Completed radiation plus concurrent temozolomide, followed by six cycles of post-radiation  temozolomide  • No evidence of progressive disease on on-study screening MRI. Residual disease or enhancement is allowed, as long as stability or response according to RANO criteria has been demonstrated compared with prior MRI  • ECOG 0-2  • Life expectancy of > 12 weeks  • Fit for further temozolomide  • Able to start study treatment within four weeks of day one of cycle six | Progressive disease on on-study screening MRI according to RANO criteria when compared with prior MRI  • Specific comorbidities or conditions (e.g. psychiatric) or concomitant medications which may interact with the administration of study treatments or procedures  • Other comorbidities or conditions that may compromise assessment of key outcomes  • No temozolomide or cranial irradiation in the last five years prior to GBM diagnosis. No other prior or concomitant therapies for glioblastoma, excluding surgery.  History of another malignancy within five years prior to registration. Patients with curatively treated carcinoma-in-situ, basal cell carcinoma of the skin, squamous cell carcinoma of the skin or superficial transitional cell carcinoma of the bladder are eligible. Patients with a history of other malignancies are eligible if they have been disease free for at least five years after definitive primary treatment  • Significant infection, including chronic active hepatitis B, hepatitis C or HIV. Testing for these is not mandatory unless clinically indicated  • Concurrent illness, including severe infection that may jeopardize the ability of the patient to undergo the procedures outlined in this protocol with reasonable safety  • Pregnancy, lactation, or inadequate contraception. Women must be post-menopausal, infertile, or use a reliable means of contraception. Women of childbearing potential must have a negative pregnancy test done within seven days prior to registration. Men must have been surgically sterilized or use a (double if required) barrier method of contraception. | • Age ≥ 75 years  • Diagnosis of metastatic castration-resistant prostate carcinoma  • Eligible for PBS-subsidised therapy with abiraterone or enzalutamide  • Suitable to receive full dose therapy  • Ability to take oral medications | • Previous systemic therapy for CRPC other than docetaxel  • Contraindication to abiraterone therapy including but not limited to:  o Uncontrolled hypertension  o Clinically significant ischemic heart disease or congestive cardiac failure  o Significant hepatic dysfunction including chronic liver disease or active viral hepatitis; ALT or AST ≥ 2.5 times upper limit of normal range or ≥ 5 times upper limit of normal in presence of liver metastases  o Pituitary or adrenal dysfunction  o Contraindication to corticosteroids  • Contraindication to enzalutamide therapy including but not limited to:  o Previous seizures or condition that confers a predisposition to seizures  o History of clinically significant neuropsychiatric event |
